# Supplementary material for: A Scalable Program for Improving Physical Activity in Older People with Dementia Including Culturally and Linguistically Diverse (CALD) Groups Who Receive Home Support: A Feasibility Study
Source: Int J Environ Res Public Health. 2023 Feb 18;20(4):3662. doi: 10.3390/ijerph20043662 (PMC9959901; doi:10.3390/ijerph20043662)
Supplement: Supplementary file 1 [file ijerph-20-03662-s001.zip › ijerph-2212729-supplementary.pdf]

Supplementary Material. Exercise manual for “Safe Functional Home Exercise” program for people with dementia

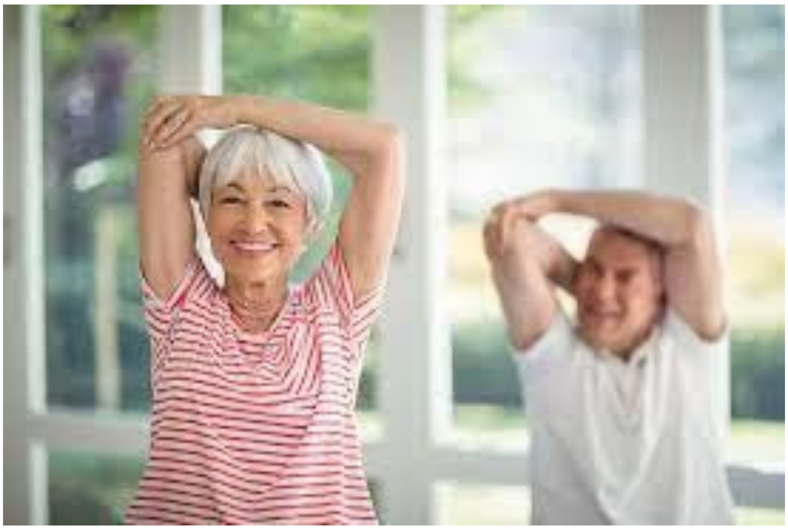

SAFE FUNCTIONAL HOME EXERCISES FOR PEOPLE WITH DEMENTIA

Manual for care support workers

A home-based, progressive strength, balance and dual task exercise program for people with dementia that can be delivered by trained care support workers

Angel Lee

Contents

Safe functional home exercises for people with dementia 3
Introduction 3
General instructions for care support worker to deliver exercise to the client and their carer 3
Start and progress the exercises 3
Engage the carer in supervising the exercises 3
Record the exercises 3
Maintenance of exercises 4
Support and trouble-shooting 4
An overview of safe functional home exercises for people with dementia 5
Benefits of safe functional home exercises for people with dementia 5
Characteristics of Level 1 and 2 exercises 5
Goals of the exercise program 6
How to start the exercise program? 6
Progression rule for exercise A, B and C 6
Progression rule for exercise D 6
A schema of safe functional home exercise for people with dementia 7
Trouble-shooting 7
Safety aspects 8
For support workers 8
For the person with dementia 8
Helping the carer to be familiar with the exercises 9
Tips for communicating and engaging the person with dementia to do exercise 9
Procedures to follow if a fall or a change in health/behaviour (relative to normal) happens 10
During exercise delivered by the care support workers 10
During exercise in between support worker visits 10
Exercise A 11
Level 1 (easy): “Bob” up and down with hand support 11
Level 2 (moderate): “Bob” up and down **without** hand support 12
Exercise B 13
Level 1 (easy): “Rock” your feet with hand support 13
Level 2 (moderate): “Rock” your feet **without** hand support 14
Exercise C 15
Level 1 (easy): Step on the “diamond” with hand support 15
Level 2 (moderate): Step on the “diamond” **without** hand support 16
Exercise D 17

Level 1 (easy): Walk and Talk at the same time with one hand support 17
Level 2 (moderate): Walk and Talk at the same time 18

Safe functional home exercises for people with dementia

Introduction

The Safe functional home exercise program was co-designed by a group of researchers, physiotherapists, staff from the home care industry and a consumer representative for older people with dementia. The aim of this exercise program is to promote physical activity levels for people with dementia, which may help to improve their physical health, functional independence and reduce their risk of falls.

The Safe functional home exercise program is a 12-week program. It has been developed such that support workers who are trained can deliver the home exercises to people with dementia and their carers. Support workers will supervise their clients to exercise for 15-20 minutes during a home care shift once weekly. The carer (e.g. family) will then supervise the person with dementia to do these exercises in between the support worker visits three days a week for 30 minutes each day.

General instructions for care support worker to deliver exercise to the client and their carer

Start and progress the exercises

- Start all exercises at Level 1 (easy).
- Progress to level 2 (moderate) if the client can perform level 1 safely and able to do 2 sets of 8-12 repetitions for that exercise with one minute rest in between the sets.
- Review exercises regularly for all clients to make sure the exercises are progressed if able.

Engage the carer in supervising the exercises

- Invite the carer to observe the exercise session during the home care shift.
- Give instructions and exercise templates to the client and carer. The target is for the client to exercise 3 days a week for 30 minutes each day over 12 weeks (the exercise can be done over several occasions in the day e.g. 3 lots of 10 minutes) under the supervision of the carer.
- Replace old exercise template(s) with new exercise template(s) if you have progressed or changed the exercises for the client and carer for the week.
- If you have **extra** capacity to supervise the client for exercises beyond your "one day a week" requirement and agreed upon by the client, carer and case manager, you can share the supervision load with the carer for the week e.g. if you supervise the exercises for one **extra** day, the carer would only need to supervise the exercises for two days in that week.

Record the exercises

- Fill in your exercise diary to record the exercise session you supervise every time. The client and carer will record the exercises they do for the week in their own exercise diary. (N.B. If

you share the supervision load with the carer beyond your "once weekly" requirement, then write these exercises that you share supervision in **their** exercise diary).

Maintenance of exercises

- If the client does not want to do the exercises on the day, you can replace the exercises with enjoyable activities e.g. light household activities that can be done standing. Try the exercises again next time.
- Obtain from the researcher the exercise goal of your client(s). Write it in your exercise diary. Referring to this regularly may help you motivate your client(s) and their carer(s) to continue to exercise.
- Obtain from the researcher the enjoyable activities of your client(s). Write them in your exercise diary. Referring to this regularly may help you find alternate physical activities for the client(s) to do if they do not want to do the exercises on the day.

Support and trouble-shooting

Contact Dr Angel Lee on 0421 136 625 or email angel.lee@monash.edu

An overview of safe functional home exercises for people with dementia

Exercise A<br>“Bob” up and down exercise	Exercise B<br>“Rock” your feet exercise	Exercise C<br>Step on the “diamond” exercise	Exercise D<br>Walk AND Talk exercise
• Level 1 (easy)<br>• Level 2 (moderate)	• Level 1 (easy)<br>• Level 2 (moderate)	• Level 1 (easy)<br>• Level 2 (moderate)	• Level 1 (easy)<br>• Level 2 (moderate)

Benefits of safe functional home exercises for people with dementia

Four exercises in the program

- Exercise A-“Bob” up and down exercise
  - Strengthen hip and knee muscles
  - These muscles are important for a person to stand up from a chair, walk and keep balance.
- Exercise B-“Rock” your feet exercise
  - Strengthen foot and ankle muscles
  - These muscles are important for balance and walking on different surfaces (e.g. even and uneven grounds).
  - Ankle movements keep a person steady to prevent a fall.
- Exercise C-Step on the “diamond” exercise
  - This exercise aims to improve balance and shifts weight in different directions.
- Exercise D-Walk AND Talk exercises
  - This exercise aims to improve ability to do these two things at the same time.
  - This ability is important for walking and balance.

Characteristics of Level 1 and 2 exercises

- Level 1 exercise is done with hand support
- Level 2 exercise is done without hand support

Goals of the exercise program

- Aim for exercise A, B, C and D to be done over the 12-week exercise program.
- Aim for high intensity i.e. doing 2 sets of 8-12 repetitions of an exercise, with one minute rest in between each set. The time to achieve this will vary between different people. Some may take several weeks.

How to start the exercise program?

- At **<u>week 1</u>**, every client starts at level 1 exercise.
- Aim to include all level 1 exercise from Exercise A, B, C and D if your client is able to do so and time permitting.

Progression rule for exercise A, B and C

- When the client can do 2 sets of 8-12 repetitions with one minute rest in between the sets of a level 1 exercise safely, progress that exercise to level 2.
- Note that the exercises can be progressed individually depending on client's ability. For example, Exercise A-level 1 can be progressed while B-level 1 and C-level 1 remained at the same level.
- Do NOT progress the exercise if the client looks unsafe or unable to do 2 sets when doing the exercise. Stay with the same level of exercise.
- Try adding difficulty to the exercise at level 1 or 2 (e.g. see pages 11 and 12) so that your client is exercising at their optimal capacity.
- Remember to review all exercises at week 5 and week 9 for progression of exercise if you have not already done so earlier.

Progression rule for exercise D

- When the client can do level 1 "talking task" with no error (i.e. if saying animal names, the client only names animals or does not repeat the same animal name) and is steady in walking, progress to level 2.
- Do NOT progress the exercise if the client looks unsafe or unable to talk without error when doing the exercise. Stay with the same level of exercise.
- Try adding difficulty to the exercise at level 1 or 2 (see pages 17 and 18) so that your client is exercising at their optimal capacity.
- Review the exercise at week 5 and week 9 for progression of exercise if you have not already done so earlier.

A schema of safe functional home exercise for people with dementia

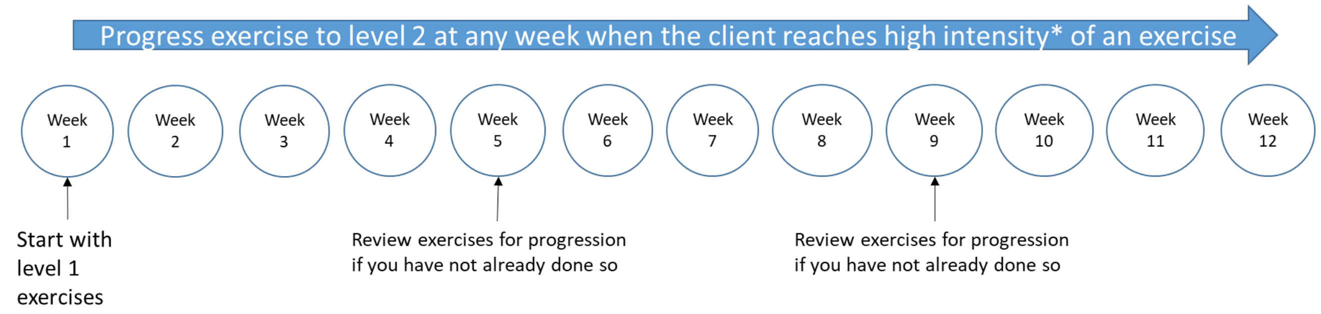

*If the client can do 2 sets of 8-12 repetitions of a level 1 exercise with one minute rest in between the sets for Exercise A, B and C OR
If client can walk safely and talk with no error for Exercise D for one minute

---

Trouble-shooting

- If the client does not want to do the exercises on the day, you can replace the exercises with enjoyable activities e.g. light household, art and craft activities or games but do them in standing. Try the exercises again the next time.
- If your client appears unsteady or unsafe after you progress them to level 2, go back to level 1 and increase the difficulty of level 1 exercise (e.g. see pages 11, 13, 15, 17). Review the exercise later for progression to level 2.
- Contact the researcher (Dr Angel Lee) for support or if you have further questions on the supervision of the exercise program.

Safety aspects

For support workers

- Stand CLOSE to the client to be able to steady the client if he/she loses balance.
- Prepare the environment for exercise so that the floor is clear of any trip or slip hazards (e.g. remove loose objects, rugs) and ensure the floor is dry. Keep pets (if any) out of the way (e.g. in a separate room).
- Offer the client with water to drink during and after the exercises, especially on warm days.
- Do not exercise with the client if:
    - They feel unwell e.g. a cold, flu, or other medical conditions.
    - They have not taken their medications as scheduled.
- Stop exercise with the client if they have any of the following symptoms during and after exercising, and do the following:
    1) Apply first aid
    2) Follow the procedure of your organisation
    3) Seek appropriate medical advice immediately

Symptoms

- Dizziness, light-headedness or feeling faint
- Chest pain, squeezing or tightness in the chest
- Difficulty breathing
- Nausea
- Excessive sweating or cold sweat
- Undue fatigue
- Shakiness
- Confusion or difficulty speaking
- Weakness
- Headache
- Irritated, moody or feeling anxious

For the person with dementia

- Wear comfortable and good fitting shoes (i.e. no loose slippers, sandals, flip flops, thongs) OR do the exercise on bare feet.
- Hold onto the kitchen bench or a sturdy chair (level 1) or stand CLOSE to the kitchen bench or a sturdy chair for higher levels of exercise (level 2).

Helping the carer to be familiar with the exercises

Encourage the carer of the person with dementia to:

- Observe you doing the exercise session.
- Note the safety information (described above) for when they are supervising exercises when the support worker is not present.
- Ask questions if they do not understand the exercises.
- Contact the researcher (Dr Angel Lee) for support or if they have further questions on the supervision of the exercise program.

Tips for communicating and engaging the person with dementia to do exercise

- Demonstrate the exercise to the client.
- Ask the client to imitate your movement.
- Give simple and consistent verbal instruction.
- Give the client time to respond to instructions.
- Reduce distraction for the client (e.g. avoid talking to the client whilst exercising or stop the client from talking whilst exercising, except for Exercise D in which the purpose is to train them to do two tasks at the same time).
- Tie the "opportunities to exercise" to the client's activity during the home care shift. Doing the exercises this way may be more "appealing" to the client as the exercises are spread out during the home care shift and incorporated into their day.
- Encourage the carer to tie the "opportunities to exercise" to the client's daily activity may be more successful for the same reason.

> *Try to be creative*
>
> When instructing the client to do an exercise, e.g. the "Bob up and down" exercise can be explained as "you are going to sit down in a chair but change your mind halfway". This may work better for some clients.
>
> *Try using music*
>
> If the client enjoys listening to music, you can play some music in the background to help make it enjoyable.

> *Examples of tagging an exercise to an activity*
>
> When the client is at the kitchen bench waiting for a cup of tea, you can supervise them to "Rock their feet"
>
> When the client is sitting watching T.V., you can ask them to stand up and supervise the "bob up and down" exercise during TV ads.

- Reward the client with praises during and after exercises (e.g. give them a high five, doing an enjoyable activity with them afterwards e.g. drinking a cup of tea together) or remind them they are getting closer to their goal.

Procedures to follow if a fall or a change in health/behaviour (relative to normal) happens

During exercise delivered by the care support workers

The exercise program has been set up by experienced physiotherapists who have used these types of exercises safely and successfully with people with dementia previously. However, if a fall or a change in health/behaviour (relative to normal) does occur, please do the following:

1) Apply first aid
2) Follow your organisation's procedure to report and manage the incident, including seeking appropriate medical advice for the client if needed.
3) You or the case manager will notify the researcher of the incident ASAP.
4) Follow your organisation's procedure (e.g. falls assessment procedure) if applicable.
5) The researcher will work with you, the case manager, client and carer to decide whether ongoing participation in the exercises is appropriate.
6) The researcher will report the event to the research ethics committee.

During exercise in between support worker visits

1) The case manager will help the carer seek medical advice for the client if needed.
2) The case manager will follow the organisation's procedure to report and manage the incident (e.g. falls assessment procedure).
3) You and/or the case manager will notify the researcher of the incident ASAP.
4) The carer will be instructed not to continue with home exercises until the researcher and/or the case manager review the exercise recommendations.
5) The researcher will report the event to the research ethics committee.

Exercise A

A. "Bob" up and down exercise (squats)

Level 1 (easy): "Bob" up and down with hand support

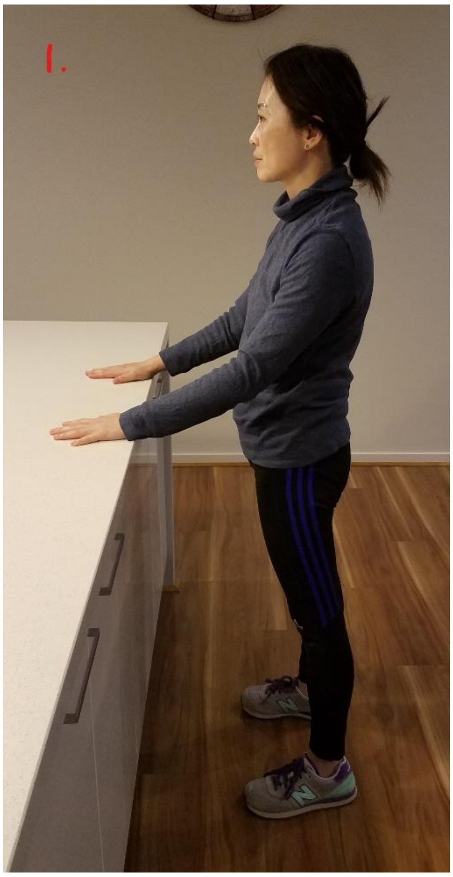

Instructions:

- Stand upright with feet shoulder width apart
- Hand support onto a steady object e.g. back of a sturdy chair or kitchen bench
- Squat down as far as you can comfortably go, hold for 1-2 seconds, and then rise up
- Aim for 8-12 repetitions for 2 sets
- "Stick" bottom out when bobbing down

The difficulty can be increased by:

- Reducing hand support e.g. changing from 2-hand support to 1-hand support
- Slowing down the squats
- Squatting deeper / lower
- Holding the squat position for more than 2 seconds before rising up
- Standing with one foot forward of the other, shoulder width apart and swap position with the other foot forward

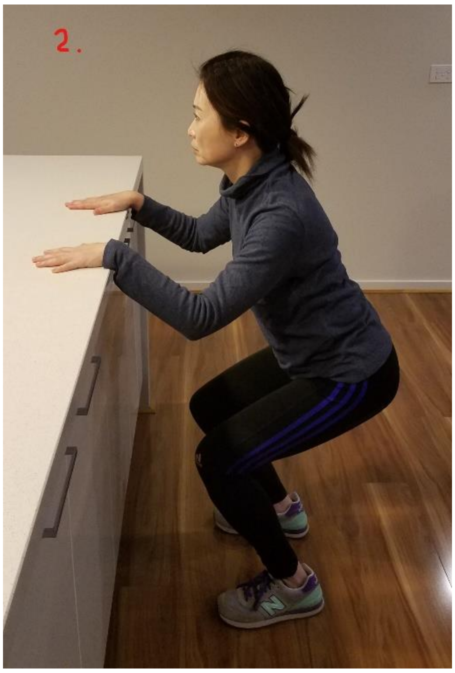

Level 2 (moderate): "Bob" up and down **without** hand support

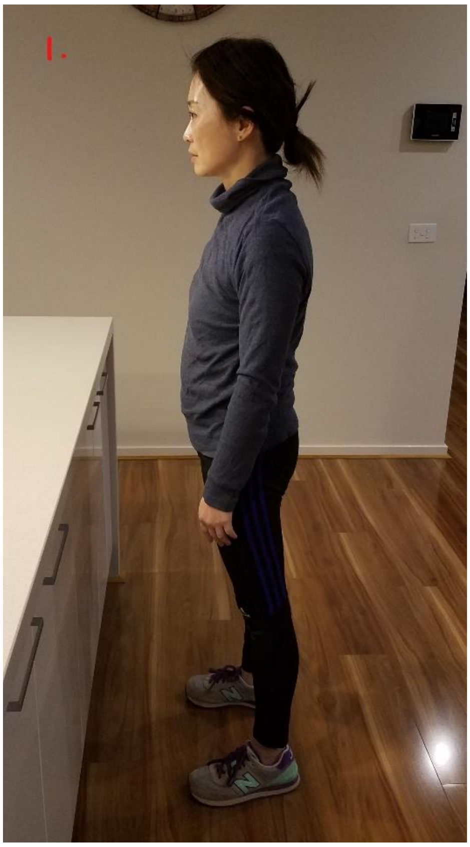

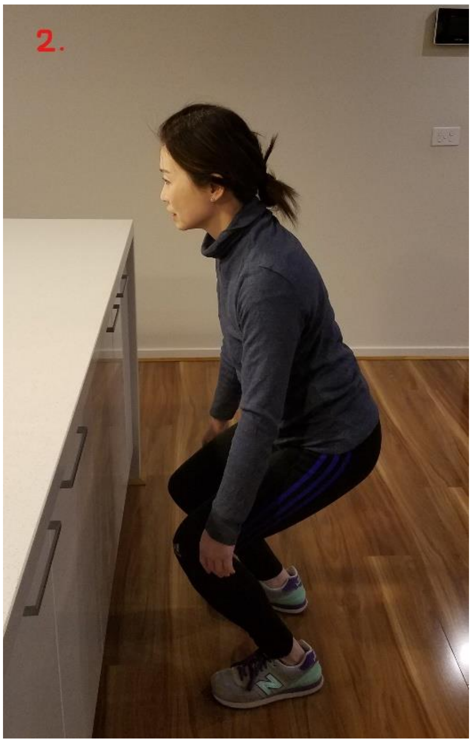

ENSURE YOU STAND CLOSELY BEHIND OR NEXT TO THE CLIENT, AND STEADY THEM IF THEY APPEAR UNSTEADY. IF TOO UNSTEADY OR NEEDING FREQUENT STEADYING, DO NOT PROGRESS TO THIS EXERCISE – GO BACK TO LEVEL 1.

Instructions:

- Stand upright with feet shoulder width apart
- Stand close to a steady object e.g. back of a sturdy chair or kitchen bench (for safety reasons in case if hand support is required)
- Squat down as far as you can comfortably go, hold for 1-2 seconds, and then rise up
- Aim for 8-12 repetitions for 2 sets
- "Stick" bottom out when bobbing down

The difficulty can be increased by:

- Slowing down the squats
- Squatting deeper / lower
- Holding the squat position for more than 2 seconds before rising up
- Standing with one foot forward of the other, shoulder width apart and swap position with the other foot forward

Exercise B

B. "Rock" your feet exercise (Heel raises)

Level 1 (easy): "Rock" your feet with hand support

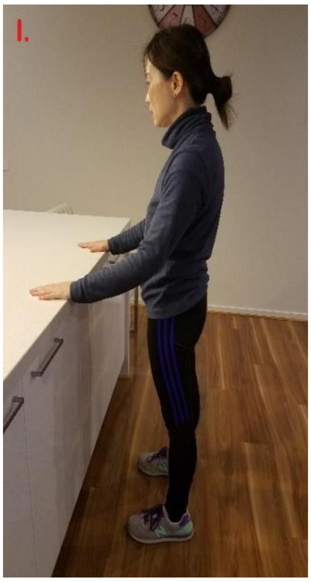

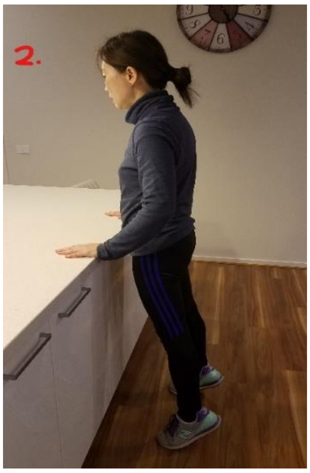

Instructions (see pictures 1, 2, 3):

- Stand upright with feet shoulder width apart
- Hand support onto a steady object e.g. back of a sturdy chair or kitchen bench
- Rise up onto toes on both feet, hold for 1-2 seconds, and then lower the heels to the floor
- Aim for 8-12 repetitions for 2 sets
- Keep hips straight when doing the exercise

The difficulty can be increased by:

- Reducing hand support e.g. changing from 2-hand support to 1-hand support
- Slowing down the heel raising / lowering
- Standing with feet less than shoulder width apart
- Adding "toe lifts" off the floor from both feet, hold for 1-2 seconds, and then lower the toes to the floor (see pictures 4 and 5)

Tips:

- Doing this exercise bare foot may be easier for some people

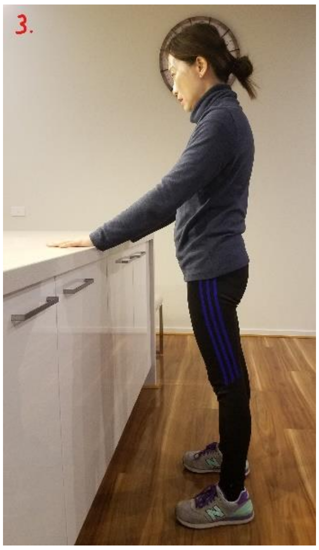

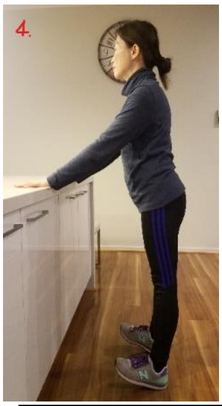

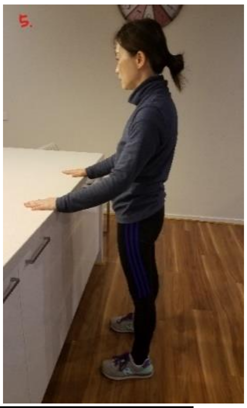

Put a sturdy chair (as a safety measure) behind the client if adding "toe lifts" exercise

Level 2 (moderate): "Rock" your feet **without** hand support

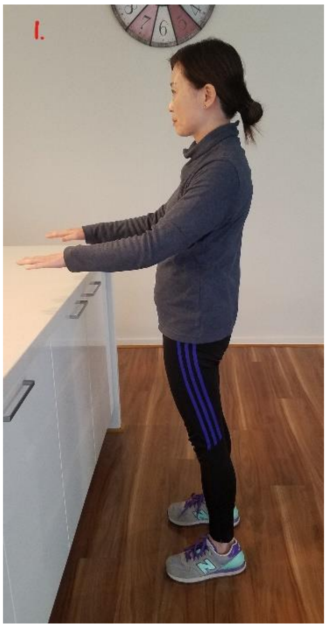

<u>ENSURE YOU STAND CLOSELY BEHIND OR NEXT TO THE CLIENT, AND STEADY THEM IF THEY APPEAR UNSTEADY. IF TOO UNSTEADY OR NEEDING FREQUENT STEADYING, DO NOT PROGRESS TO THIS EXERCISE – GO BACK TO LEVEL 1.</u>

<u>Instructions (see pictures 1, 2, 3):</u>

- Stand upright with feet shoulder width apart
- Stand close to a steady object e.g. back of a sturdy chair or kitchen bench
- Hands can hover over the steady object for safety reasons if required (see pictures 1, 2, 3)

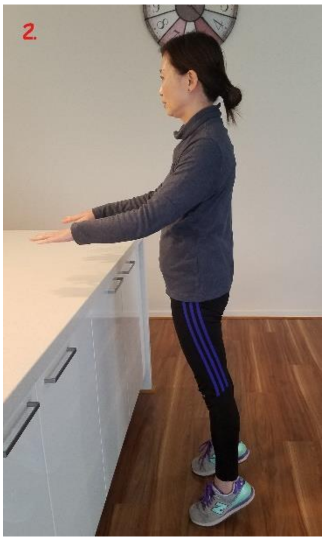

- Rise up onto toes on both feet (no hand support), hold for 1-2 seconds, and then lower the heels to the floor
- Aim for 8-12 repetitions for 2 sets
- Keep hips straight when doing the exercise

<u>The difficulty can be increased by:</u>

- Slowing down the heel raising / lowering
- Standing with feet less than shoulder width apart
- Adding "toe lifts" off the floor from both feet, hold for 1-2 seconds, and then lower the toes to the floor (see pictures 4 and 5)

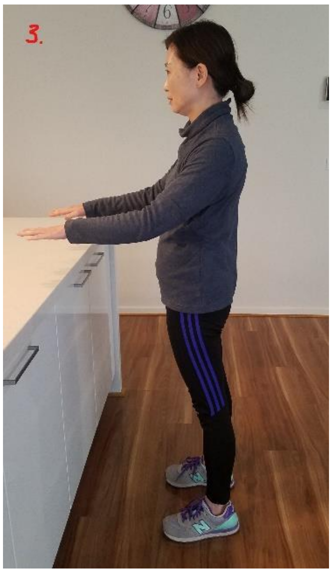

<u>Tips:</u>

- Doing this exercise bare foot may be easier for some people

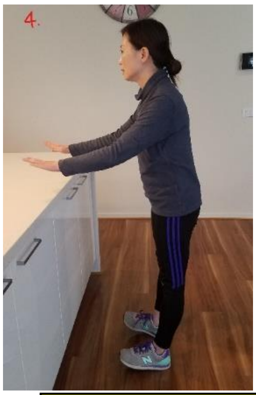

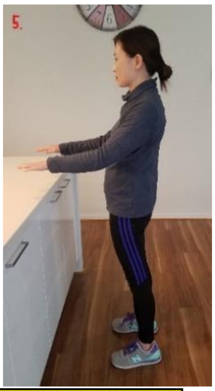

Put a sturdy chair (as a safety measure) behind the client if adding "toe lifts" exercise

Exercise C

C. Step on the “diamond”

Level 1 (easy): Step on the “diamond” with hand support

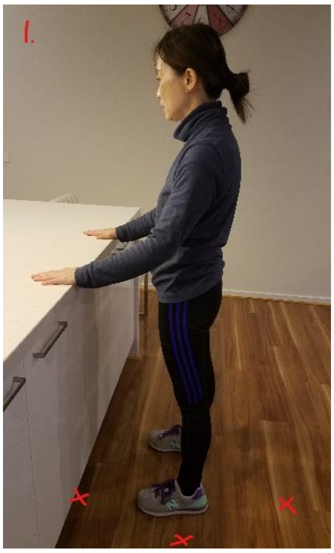

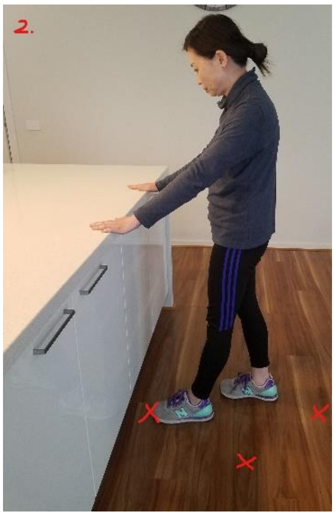

Instructions:

- Stand upright with feet shoulder width apart
- Hand support onto a steady object e.g. back of a sturdy chair or kitchen bench
- Take a step forward with the left foot (to the front point of the diamond ✗), then
- Take a step to the side with the left foot (to the side point of the diamond ✗, then
- Take a step to the back with the left foot (to the rear point of the diamond ✗), then
- Return to the start position
- Swap foot. Repeat the stepping pattern with the right foot.
- Aim for 8-12 repetitions for 2 sets

The difficulty can be increased by:

- Taking a bigger step to the front, side and rear points of the diamond
- Slowing down the step movements so that they spend more time standing on one leg

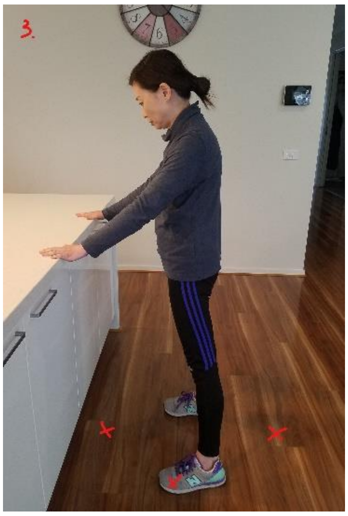

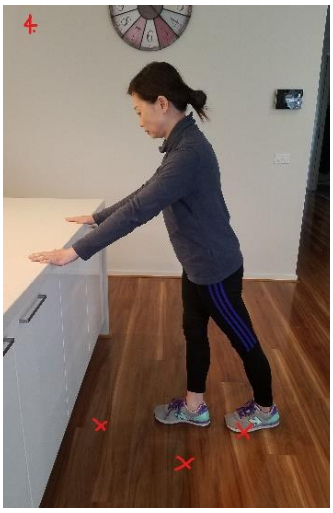

- Standing with feet less than shoulder width apart
- Reducing hand support e.g. changing from 2-hand support to 1-hand support

Tips:

- Allow the client to take a step to one ✗ point if they cannot step out to all the ✗ points

Level 2 (moderate): Step on the "diamond" **without** hand support

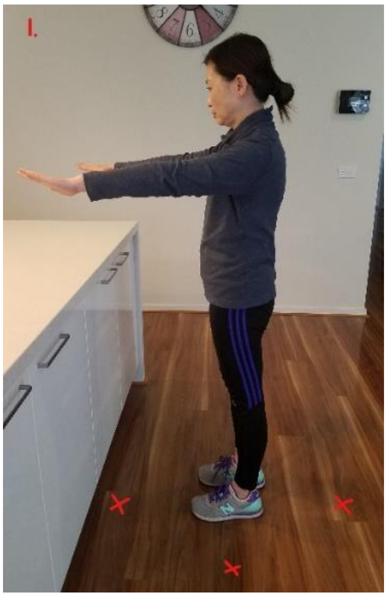

<u>ENSURE YOU STAND CLOSELY NEXT TO THE CLIENT ON THE OPPOSITE SIDE, AND STEADY THEM IF THEY APPEAR UNSTEADY. IF TOO UNSTEADY OR NEEDING FREQUENT STEADYING, DO NOT PROGRESS TO THIS EXERCISE – GO BACK TO LEVEL 1</u>

<u>Instructions:</u>

- Stand upright with feet shoulder width apart
- Stand close to a steady object e.g. back of a sturdy chair or kitchen bench
- Hands can hover over the steady object for safety reasons if required (see pictures 1, 2, 3, 4).
- Take a step forward with the left foot (to the front point of the diamond ✗), then
- Take a step to the side with the left foot (to the side point of the diamond ✗, then
- Take a step to the back with the left foot (to the rear point of the diamond ✗), then
- Return to the start position
- Swap foot. Repeat the stepping pattern with the right foot.
- Aim for 8-12 repetitions for 2 sets

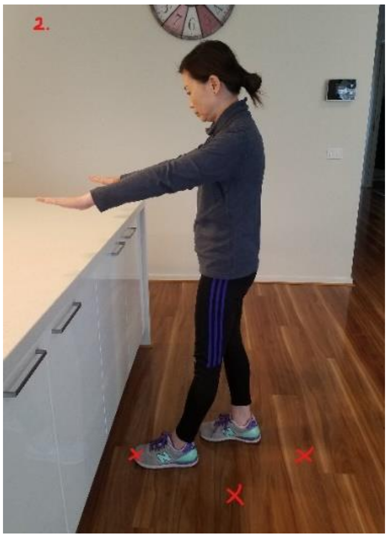

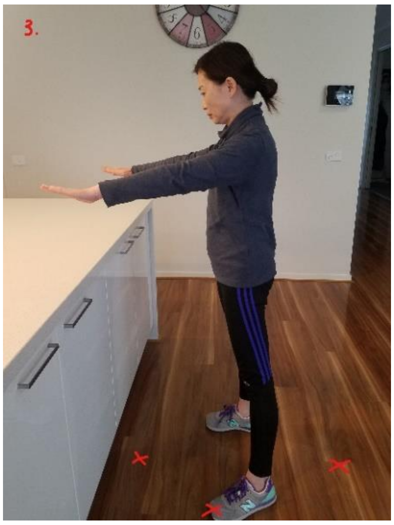

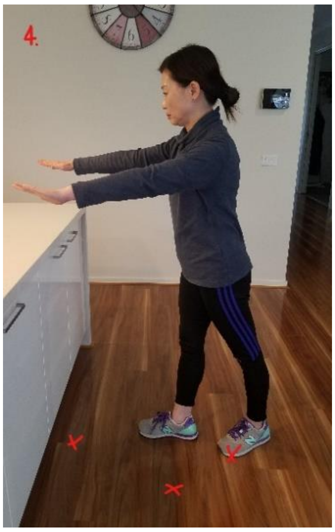

<u>The difficulty can be increased by:</u>

- Taking a bigger step to the front, side and rear points of the diamond
- Slowing down the step movements so that they spend more time standing on one leg
- Standing with feet less than shoulder width apart

<u>Tips:</u>

- Allow the client to take a step to one ✗ point if they cannot step out to all the ✗ points

Exercise D

D. Walk and Talk exercise

Level 1 (easy): Walk and Talk at the same time with one hand support

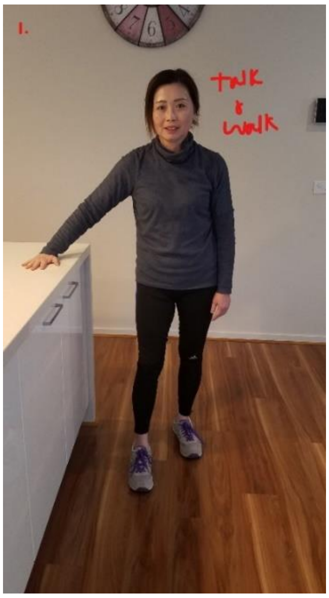

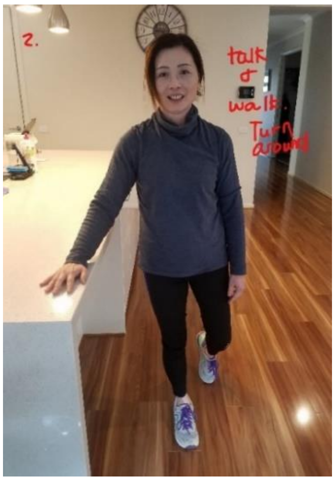

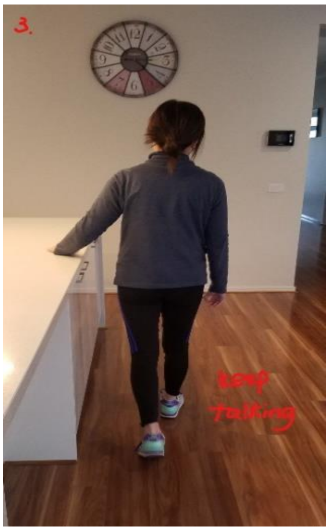

Instructions:

- Walk forward alongside the kitchen bench with one hand supported on the kitchen bench.
- Start walking from one end of the bench to the other end. Turn around to return to the start
- Walk for 1 minute
- Talk whilst walking. Example topics:
    - Carry out and maintain a daily conversation
    - The client says different fruit and vegetable names aloud
    - The client says different animal names aloud
- Aim for no error in the "talking task" (i.e. if saying animal names, the client does not name things other than animals or repeat the same animal name) and steadiness in walking

The difficulty can be increased by:

- Do a more challenging talking task e.g. counting backwards aloud by 2's (harder if by 3's) from 100

Level 2 (moderate): Walk and Talk at the same time

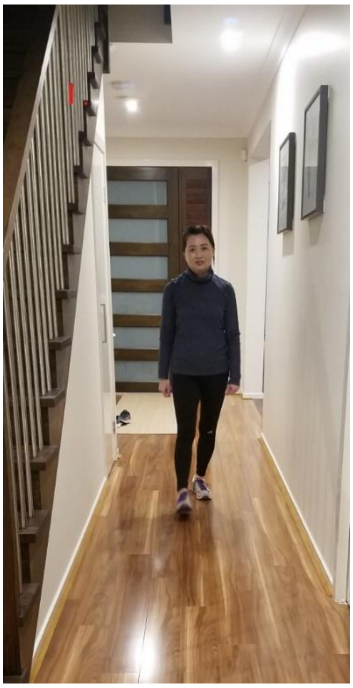

ENSURE YOU WALK CLOSELY BEHIND THE CLIENT, AND STEADY THEM IF THEY APPEAR UNSTEADY. IF TOO UNSTEADY OR NEEDING FREQUENT STEADYING, DO NOT PROGRESS TO THIS EXERCISE – GO BACK TO LEVEL 1.

- Allow walking aid if the client normally walks with one

Instructions:

- Walk forward in a straight path (e.g. a corridor), turn around to return to the start
- Walk for 1 minute
- Talk whilst walking. Example topics:
  - Carry out and maintain a daily conversation
  - The client says different fruit and vegetable names aloud
  - The client says different animal names aloud
- Aim for no error in the "talking task" (i.e. if saying animal names, the client does not name things other than animals or repeat the same animal name) and steadiness in walking

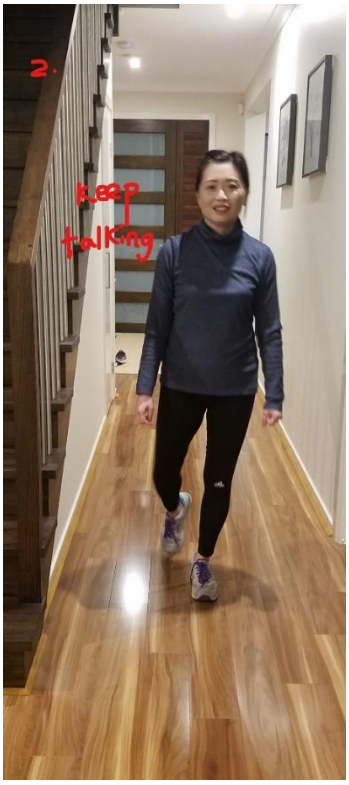

The difficulty can be increased by:

- Do a more challenging talking task e.g. counting backwards aloud by 2's (harder if by 3's) from 100
